# Open-source Raman spectra of chemical compounds for active pharmaceutical ingredient development

**DOI:** 10.1038/s41597-025-04848-6

**Published:** 2025-03-24

**Authors:** Aaron R. Flanagan, Frank G. Glavin

**Affiliations:** School of Computer Science, University of Galway, Galway City, Co. Galway H91 FYH2 Ireland

**Keywords:** Infrared spectroscopy, Raman spectroscopy, Drug development

## Abstract

Raman spectroscopy is utilised extensively in pharmaceutical analysis for tasks such as drug discovery, quality control and active pharmaceutical ingredient (API) development. Despite this, access to open-source Raman spectral datasets for modelling and analysis is often a challenge. In laboratory settings, small spectral libraries are typically compiled for one-shot identification of intermediates or unknown chemicals, which restricts availability to comprehensive and high-quality reference data. In this work, we introduce a new open-source Raman dataset consisting of pure chemical compounds commonly employed in the development of APIs. By curating and publishing this dataset, we aim to provide the scientific community with access to high-quality, reusable data. Containing 3,510 samples spanning 32 compounds, this data can be utilised for referencing and can potentially facilitate in the development of more accurate and generalisable calibration models when access to reference data is limited.

## Background & Summary

The Raman spectrum is a function of the inelastic scattering of light, specifically from molecular vibrations and rotations within a sample^1,2^. When an excitation source, typically a monochromatic laser, illuminates the sample, molecules that are sensitive to changes in polarisability infrequently undergo an energy exchange with the incident radiation. This interaction induces a transition, shifting the molecules to a higher virtual energy state, and almost instantaneously, the molecules relax back to their ground state, scattering the radiation. The back-scattered light is filtered according to frequency when compared directly with the original light source. Lower (Stokes) or higher (anti-Stokes) frequency light is directed onto a detector, such as a charge-coupled device (CCD), producing the Raman spectrum. Each wavelength in the spectrum is associated with a shift in frequency, measured in reciprocal centimetres (cm^-1^), and referred to as the Raman shift. Each peak in the Raman spectrum corresponds to a vibrational or rotational mode, associated with a specific molecular transition. The magnitude of the peaks, presented as intensity in arbitrary units, correspond to an absolute count rate of observed events at that wavelength. The spectrum represents a fingerprint that enables identification of the sample via its molecular composition. It is typically divided among three distinct regions: (i) the low wavenumber region (100–200 cm^-1^), (ii) the fingerprint region ranging upwards of 1800–2000 cm^-1^, and (iii) the high wavenumber region corresponding to many frequent organic carbon-based vibrations.

Raman-based technologies^3–5^ have been employed in the field of pharmaceuticals for tasks such as the identification of tablets^6^, drug discovery^7,8^, and as a process analytical technology (PAT) tool in active pharmaceutical ingredient (API) manufacturing^9,10^. While Raman is fast, efficient, and non-destructive, a common challenge is the manual effort required for sample preparation and the identification of unknown chemical substances. To address this issue, spectral databases for sample referencing have been created, and machine learning algorithms have been investigated to improve analysis and automate these tasks^11^. However, another significant challenge for both solutions is data scarcity. Access to extensive open-source databases is often unavailable or expensive to create, and robust calibration typically demands large quantities of data that comprehensively cover as much variation in the problem domain as possible.

Many open-source databases provide single or limited sample sets of experimental Raman spectra. Popular examples include the RRUFF mineral database^12^ and the Raman Open Database^13^, both of which offer crystallographic information. Additionally, computational spectral libraries such as the WURM project^14^ and the Computational 2D Materials Database (C2DB)^15^ serve as efficient supplementary resources for materials identification and classification. Alternatively, the subscription-based Wiley KnowItAll Raman Spectral Library (https://sciencesolutions.wiley.com/solutions/technique/raman/knowitall-raman-collection) provides an extensive reference database, though it remains proprietary.

**Fig. 1:**
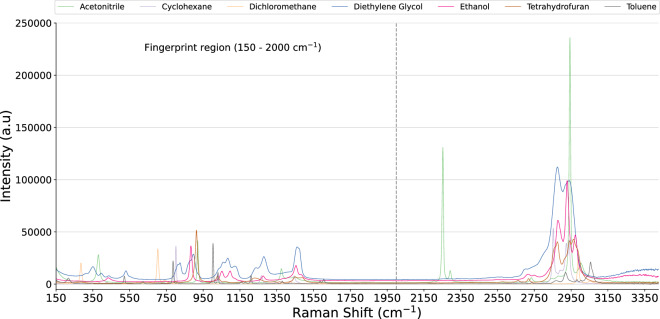
Representative Raman spectra of seven common chemicals used in API development, with the fingerprint region highlighted.

In today’s data-driven era, open-source libraries with comprehensive, high-quality data are crucial for spectral analysis and modelling. Research efforts have been made to address this need^16–18^, and to investigate computational methods in the absence of experimental data^19–21^. Inspired by this objective, we introduce a new open-source Raman spectral dataset comprising 3,510 spectra from 32 chemical substances. This dataset includes organic solvents and reagents commonly used in API development, aiming to provide accessible, high-quality data for the scientific community. Figure 1 presents representative samples for seven of the products. Its potential applications range from sample analysis and modelling to broader use cases, such as training new researchers in preprocessing techniques, peak identification in unknowns, or foundational pre-training of machine learning models.

## Methods

### Experimental Configurations

**Fig. 2:**
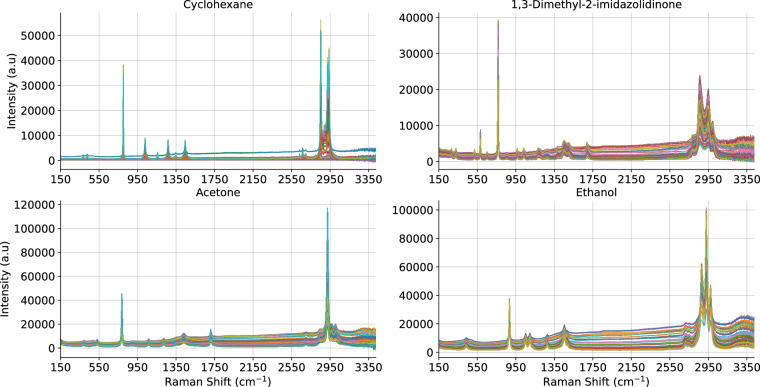
Example batches of four compounds to demonstrate the inter-class variation.

The system employed in this study was an *Endress+Hauser* (formerly *Kaiser Optical Systems*) Raman Rxn2 analyser (https://www.ie.endress.com/en/field-instruments-overview/optical-analysis-product-overview/raman-rxn2-analyzer?t.tabId=product-overview) powered by Kaiser Raman technology. This included a three-quarter inch non-contact Rxn-10 probe fitted with a 10x objective lens. The excitation wavelength was 785 nm with an average spectral resolution of 1 cm^-1^ covering 3276 wavelengths in the range of 150 to 3425 cm^-1^. The spectral acquisition and experimental details were configured via *Mettler Toledo* iC Raman^TM^ 4.1 software (https://www.mt.com/int/en/home/products/L1_AutochemProducts/automated-reactor-in-situ-analysis-software/ic-raman-instruments.html). Prior to scanning each product, the probe was focused using the iC Raman^TM^ pixel fill function. The pixel fill function provides a measure of the absolute intensity count rate at any wavelength, given the probes current distance from the sample^22^. Per the iC Raman^TM^ software guidelines, the optimal pixel fill ranged between 40 – 70%, with excess of 70% saturating the detector. In this work, the pixel fill was optimised to obtain the best possible resolution, such that the thresholds were set between 50–70% depending on the strength of the sample as a Raman scatterer. Figure 2 presents example batches for 4 of the compounds to demonstrate the variability captured in each group.

### Materials

Thirty-two commercial solvents and reagents were collected using the Raman Rxn2 analyser with iC Raman^TM^. Table 1 presents the information on the products, which includes the product name, percentage purity (assay), exposure time measured in seconds, pixel fill calibration percentage, and number of samples available after curation. The samples presented in this work are used at full concentration and published without further purification or preprocessing. Most products have an assay above 99%, but trace impurities from synthesis may still be present. Impurities in pure chemical compounds can come from various sources including starting material contamination, residual solvents, environmental causes, and storage degradation^23^. With regard to starting material contamination and residual solvents, these impurities are unavoidable if they are present in the products provided by the suppliers. To address this, only products tested and successfully applied in API developments were sampled. To avoid introducing storage related impurities, expiration protocols were followed and each product was stored in an appropriate laboratory grade storage solution respective to the product, such as flammable, acid and corrosive, or ventilated chemical storage cabinets. Lastly, to avoid environmental causes, each product was sampled in a fume hood, then immediately sealed in a 4 mL amber glass vial to prevent contamination.

**Table 1:** Experimental details of data acquisition.

Name	Assay	Exposure	Pixel Fill	Samples
1,3-Dimethyl-2-imidazolidinone	≥ 99.0	3	50	109
2-Propanol	≥ 99.8	5	50	109
2,2-Dimethoxypropane	≥ 98.0	5	60	107
4-Methyl-2-pentanone	≥ 99.5	15	50	100
Acetic acid glacial	≥ 99.8	7.5	60	113
Acetone	≥ 99.8	7	73	120
Acetonitrile	≥ 99.9	10	50	104
Benzaldehyde	≥ 99.0	2	68	121
Benzyl bromide	≥ 98.0	2	73	112
Butyl acetate	≥ 99.7	20	51	106
Chloroform	≥ 99.8	2	70	122
Cyclohexane	≥ 99.8	2	70	145
Dichloromethane	≥ 99.5	5	55	111
Diethyl malonate	≥ 99.0	30	63	100
Diethylamine	≥ 99.5	15	60	107
Diethylene glycol	≥ 99.0	30	50	105
Dimethyl sulfoxide	≥ 99.8	2	60	105
Ethanol	≥ 99.9	10	56	109
Ethyl acetate	≥ 99.8	10	50	110
Ethylene glycol	≥ 99.0	15	70	101
Formic acid	≥ 98.0	20	55	110
Isobutylamine	≥ 99.0	20	60	104
Methanol	≥ 99.8	15	50	101
Methyl isobutyl ketone	≥ 99.5	20	55	105
N,N-Dimethylformamide	≥ 99.8	10	40	105
n-Heptane	≥ 95.0	45	68	103
n-Hexane	≥ 98.0	30	55	102
Propyl acetate	≥ 99.0	20	63	107
tert-Butanol	≥ 99.7	3	51	113
tert-Butyl methyl ether	≥ 99.8	3	60	117
Tetrahydrofuran	≥ 99.9	5	70	100
Toluene	≥ 99.9	2	67	127

### Data Acquisition

After sampling, the 4 mL amber vial was placed into a static analysis chamber fitted with the Rxn-10 probe. The empty amber vials were scanned and analysed to confirm that scattering effects and other artefacts were not introduced during acquisition. Each spectrum in the dataset corresponds to a single manual scan captured over the specified exposure time. The Rxn-10 probe was moved laterally and by twisting it around its cylindrical axis to simulate various optical path lengths and positions. Automatic pre-treatment was applied via the iC Raman^TM^ experimental configurations. This included dark noise subtraction, cosmic ray filtering, and intensity correction. No other spectral preprocessing was applied after collection. The raw spectral data were then exported as individual SPC files and formatted into a CSV file for easy access.

### Raman Bands

Tables 2–3 present an overview of the active Raman bands for each compound. The Raman bands highlight the regions where minor or major peaks are present, indicating Raman activity. Distinct peaks, indicating a high quantity of activity, are highlighted in bold, along with other minor peaks that were detected. It is important to note that there is no significant or statistical difference between the groups; the tables are divided into two separate groups to achieve cleanliness and compactness.

**Table 2:** Group 1 product names and Raman active bands (in cm^−1^).

Product	Raman bands
1,3-Dimethyl-2-imidazolidinone	279, 326, 520, **581,** **766**, 964, 1031, 1183, 1452, 1494, 1669, 2785, **2860,** **2951**, 2995
2-Propanol	373, 429, 489, **820,** **953**, 1132, 1342, **1452**, 2721, **2882,** **2920,** **2975**
2,2-Dimethoxypropane	263, 399, 552, **583,** **731**, 828, 927, 1081, **1442**, 2727, **2830,** **2947,** **2996**
4-Methyl-2-pentanone	318, 419, **594**, 785, 817, 959, 1124, **1451,** **1464**, 1711, **2874,** **2920**, 2962
Acetic acid	446, 622, **894**, 1429, 1668, **2943,** **2994**
Acetone	392, 531, **788**, 1067, 1222, 1431, **1709**, 2698, 2850, **2924**, 2967, 3007
Acetonitrile	**380,** **920**, 1375, **2253**, 2293, 2732, **2994**, 3004
Benzaldehyde	225, **439**, 616, 650, **829,** **1001**, 1166, **1204,** **1598**, 1653, **1698**, 2741, 2824, 2976, 3012, **3067**
Benzyl bromide	**237,** **454**, 548, **605**, 759, 813, **1002**, 1029, 1157, 1180, **1227,** **1602,** **2971,** **3059**
Butyl acetate	**307,** **634,** **839**, 919, 1031, 1066, **1032,** **1451,** **1738**, 2738, **2877**, 2917, **2940**
Chloroform	**261,** **366,** **668**, 758, 1216, **3019**
Cyclohexane	384, 426, **802,** **1028**, 1158, **1266**, 1347, **1444**, 2633, 2665, 2698, 2800, **2853**, 2897, **2939**
Dichloromethane	**286,** **703**, 741, 1156, **1423,** **2988**, 3056
Diethyl malonate	**339**, 377, 573, 677, 789, **847**, 934, 961, 1034, **1115**, 1273, 1302, 1416, **1455,** **1749**, 2728, 2777, 2878, **2942,** **2976**
Diethylamine	370, **426**, 496, **866**, 928, **1047**, 1139, 1195, 1256, **1456**, 2644, 2720, 2748, 2775, **2815,** **2872,** **2925**, 2970, **3317**
Diethylene glycol	**351**, 295, 436, **530,** **824,** **897**, 1059, **1084**, 1121, 1238, **1280,** **1461**, 2703, **2876**, 2941

**Table 3:** Group 2 product names and Raman active bands (in cm^−1^).

Product	Raman bands
Dimethyl sulfoxide	307, **334**, 383, **669**, 699, 954, **1044,** **1419,** **2913,** **2997**
Ethanol	443, **883,** **1052,** **1096**, 1276, **1454**, 2718, **2878,** **2929**, 2973
Ethyl acetate	**379,** **634**, 787, **847**, 918, 939, 1002, 1047, **1115,** **1454,** **1736**, 2714, 2879, **2941**, 2979
Ethylene glycol	347, **481,** **865**, 1046, **1091,** **1271,** **1462**, 2722, **2881,** **2937**
Formic acid	186, **679**, 1061, **1204,** **1398,** **1660**, 2782, **2963**
Isobutylamine	362, **483**, 773, **798**, 957, 1063, 1131, 1172, 1339, **1461**, 2719, 2774, **2870**, 2907, 2928, **2957,** **3328**
Methanol	**1034**, 1110, 1156, **1452,** **2835,** **2944**
Methyl isobutyl ketone	178, **318**, 419, **594,** **786**, 817, 959, **1124**, 1172, 1334, **1464,** **1711**, 2722, 2764, 2874, 2921, 2962
N,N-Dimethylformamide	**318,** **355,** **405,** **659,** **866,** **1092,** **1407**, 1440, **1661**, 2808, 2869, **2932**, 2997
n-Heptane	**309**, 356, 395, 777, **839,** **902**, 1045, **1079,** **1138,** **1302,** **1455,** **2732,** **2875**, 2938
n-Hexane	317, **371**, 402, 454, **823,** **892**, 1005, 1039, 1080, 1141, **1303,** **1456**, 2671, 2733, **2876,** **2938**
Propyl acetate	309, **348**, 413, **632,** **837,** **894**, 967, 1045, 1280, **1454,** **1739**, 2742, **2882,** **2941**
tert-Butanol	**347**, 476, **751,** **916**, 1026, **1209,** **1240,** **1454**, 2713, 2864, **2920,** **2976**
tert-Butyl methyl ether	285, 372, **509,** **726,** **853,** **916**, 1020, 1086, 1177, 1234, 1264, **1446**, 2711, **2829,** **2929,** **2978**
Tetrahydrofuran	186, **679**, 1061, **1204,** **1398,** **1660**, 2782, **2963**
Toluene	**217,** **522**, 622, **786,** **1003,** **1030**, 1156, 1179, **1210**, 1380, 1586, **1605**, 2868, **2920**, 2982, **3056**

### Handling Spectral Offsets

The raw spectra exhibit fluorescence and linear baseline offsets, requiring preprocessing before applying it for tasks such as calibration. The last region of wavenumbers (≥ 3150 cm^-1^) do not correspond to Raman activity, and it is recommended to crop this region. This will limit the effect of outliers on the resulting shape and magnitude of the spectrum during baseline correction and data scaling.

**Fig. 3:**
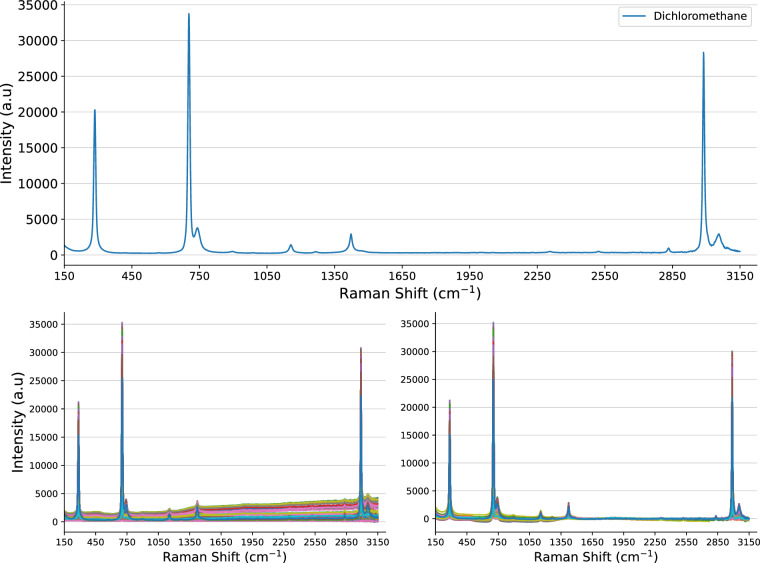
Plot demonstrating a two-point baseline correction applied to dichloromethane. This includes the best sample for reference (top), with the raw sample batch (bottom-left), and baseline corrected batch (bottom-right).

With regard to baseline correction, preliminary tests indicate a simple two-point correction algorithm effectively reduces the linear offset, as demonstrated in Fig. 3. This method involves selecting the first and last wavelengths in the spectrum, drawing a linear line between these points, and subtracting it from the spectrum. While this approach efficiently handles basic linear offsets, more advanced algorithms may be necessary for fine-scale corrections. Examples include Fourier and wavelet transforms (WT), Savitzky-Golay (SG) filters, asymmetric least squares (ALS), and multiplicative signal correction (MSC)^24^. Our tests show mixed results for MSC when using the mean and when selecting the best sample as reference, with the two-point correction often proving more effective. However, further fine-tuning and testing may yield improved results. These findings suggest that exploring new, potentially more robust techniques, is required to effectively clean the data.

**Fig. 4:**
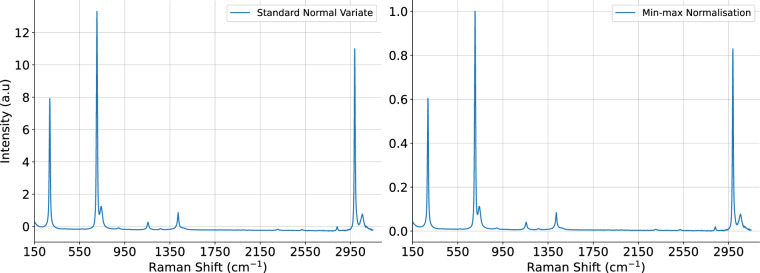
Two-point baseline corrected spectrum of dichloromethane when scaled via SNV (left) and min-max normalisation (right).

For data scaling, we recommend the standard normal variate (SNV) or min-max normalisation techniques^24^. The SNV algorithm is applied per sample (rows), and the scale will be relative to the spectrum. Min-max normalisation scales the data between 0 - 1 for easier comparison. It is typically calculated over the wavelengths (columns) of the full dataset; however, we advise that it is also applied per sample to limit the effect of outliers. Figure 4 demonstrates the scaling methods on a two-point baseline corrected sample of dichloromethane from Fig. 3.

## Data Records

The experimental data presented in this work is deposited in a public repository hosted by *Figshare*^25^. The raw spectra are provided in a file named *raman_spectra_api_compounds.csv*, which can be processed by any programming language or software capable of handling CSV files. The data is organised as an *N* × *M* matrix, where *N* represents the number of samples, and *M* corresponds to the 3276 wavelengths. Each row in the matrix corresponds to a single sample, with the respective target label located in the last column. The first row serves as the header, containing wavenumbers from 150 - 3245 cm^-1^ with a 1 cm^-1^ resolution. For easy identification and analysis, the spectra are sorted into groups by compound. The best representation, with the highest resolution, is the first sample in each product batch. If the data is to be used for calibration, we recommend shuffling it first to avoid any unintentional biases.

The online repository^25^ also includes an XLSX file which contains additional information about the products used to collect the data. The file is named *API_Product_Information.xlsx*, and it contains the information found in Tables 1–3, along with the supplier and product name, the product’s role (as a solvent, reagent, or both), its application (as an intermediate or reactant), and molecular formula.

## Technical Validation

### System Stability

To ensure reliable and reproducible results, the system was calibrated using an *Endress+Hauser* Raman calibration accessory (https://www.ie.endress.com/en/field-instruments-overview/optical-analysis-product-overview/raman-calibration-accessory?t.tabId=product-overview), which included both wavelength and intensity calibrations. Following the manufacturer’s recommendations, the system was granted a 120-minute warm-up period each day before use. This was followed by automatic intensity and wavelength calibrations. The system typically operated within a temperature range of 15–30 °C, while the detector was maintained at a constant temperature of -40 °C. During operation, the system consumed an average power of 120 W, with a maximum output of 400 W. Cyclohexane was used as the reference standard for both initial calibration and for monitoring day-to-day system variations. A fresh sample was prepared, focused using the pixel fill function, and scanned after auto-calibration, as well as before each new compound.

### Data Reliability

**Fig. 5:**
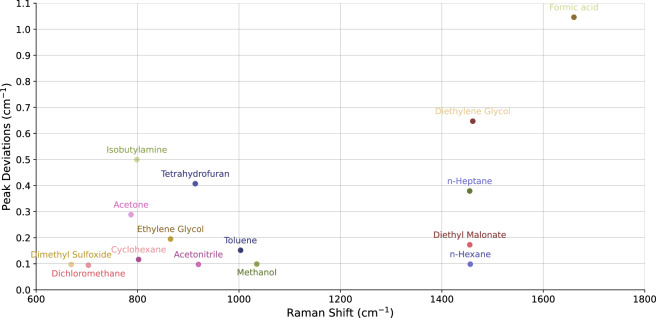
Observed standard deviations of mean peak maxima positions for fifteen of the thirty-two products.

To further assess the reliability of the Raman Rxn2 instrument and the products, we conducted measurement repeatability tests after data acquisition to validate the stability of peak positions. For each product, we manually defined a peak region that includes the wavenumber corresponding to the maximum intensity observed in the fingerprint region (≤ 2000 cm^-1^) of the spectrum. The most intense peak, typically corresponding to the strongest Raman scatterer, is less susceptible to background interference. Each peak region spans 200 cm^-1^, centred around the maximum intensity peak. For example, the peak region for 1,3-dimethyl-2-imidazolidinone was defined as 666–866 cm^-1^, with the maximum intensity peak at 766 cm^-1^. This approach ensures that small shifts in detected peak positions do not significantly affect the measurements, while also accounting for possible shifts due to broad baselines from fluorescence or other background effects. Within each 200 cm^-1^ range, we calculated the mean and standard deviation of the maximum intensity peak for each product. The results demonstrate excellent repeatability, with over 50% of the products showing no deviation from the average peak position. Figure 5 presents the results only for products with observed deviations, while those with a deviation of 0 are omitted. The standard deviations observed were less than 1 cm^-1^, except for formic acid, which showed a deviation of 1.0462.

## Data Availability

The data wrangling and analysis for this work was conducted using Python v3.11.8 (https://www.python.org/downloads/release/python-3118/). The raw data was curated using Python libraries spc_spectra v0.4.0 (https://pypi.org/project/spc_spectra/) and pyspectra v0.0.1.2 (https://pypi.org/project/pyspectra/) to load the data from the iC Raman^TM^ SPC file format. The code for generating the figures, identifying the active Raman bands, performing preprocessing, and calculating the mean peak positions are available as Juypter notebooks, located in the *code* folder in the online repository^25^.
